# 

**Published:** 1852-01

**Authors:** 


### NEW ARRANGEMENT.

At the close of the present volume of the Journal, it will pass entirely out of the hands of the present Editor and Proprietor into those of our former colleague, Prof. Herrick, with whose writings our readers are already familiar. But the accounts of the establishment that have already accrued and such as accrue with the present volume, remain in the hands of the present editor as his own private and individual property. We have a few words to say, therefore, upon the subject of arrearages at the present time, that we may be clearly understood, and that our friends who have not paid up may have an opportunity of saving one dollar for each year they may be in arrears to the Journal. By turning to the prospectus of the two last and the present volumes of the Journal, it will be seen that the terms are *three* dollars a year unless paid before the close of the volume. At the close of this volume, the last number of which will issue on the first of March next, the accounts of all delinquent subscribers will be placed in the hands of an attorney for collection, and the three dollars a year for the last three volumes will invariably be required. It will cost us at least that much more to collect them than it ought.

Now we offer to take two dollars a year up to the close of the present volume as a full liquidation of our claims against all regular subscribers who pay up before the close of this volume. It is a small amount to any one of our subscribers and may as well be paid at one time as another, and we hope to hear no complaints about paying the extra dollar per volume from those who do not see fit to take up with this offer.

### RECEIPTS FOR THE JOURNAL FOR NONEMBER AND DECM'BR.

| | |
|---|---|
| **ILLINOIS.** | |
| Dr. A. W. Benton, | $2 00 |
| Samuel Thompson, | 2 00 |
| —— Rosenberge, | 2 00 |
| W. S. Knight, | 1 00 |
| G. W. Caruthers, | 2 00 |
| Estate of Dr. Boice, | 2 00 |
| Dr. B. Tenney, | 2 00 |
| E. McArthur, | 4 00 |
| P. Kirwin, | 4 00 |
| A. L. McArthur, | 2 00 |
| W. W. Cavarly, | 4 00 |
| G. W. Rittell, | 2 00 |
| P. W. Randle, | 2 09 |
| **INDIANA.** | |
| Dr. W. Andrews, | 5 00 |
| W. Bamford, | 4 00 |
| G. W. Shelden, | 5 00 |
| C. V. Jones, | 2 00 |
| R. C. Moore, | 2 00 |
| L. H. Kennedy, | 4 00 |
| D. McKyssick, | 2 00 |
| Dr. Jno. Lewis, | 2 00 |
| T. D. Brown, | 2 00 |
| M. Bell, | 2 00 |
| J. E. Lloyd, | 2 00 |
| J. McCorkle, | 2 00 |
| **WISCONSIN.** | |
| Dr. C. B. Pearson, | $2 00 |
| A. Castleman, | 2 00 |
| C. H. Orten, | 1 50 |
| C. C. Robinson, | 5 00 |
| Rowley Morris, | 2 00 |
| **MICHIGAN.** | |
| Dr. John McLean, | $2 00 |
| Isaac Suyder, | 2 00 |
| B. Bu gham, | 2 00 |
| J. B. W. Lewis, | 2 00 |
| N. D. Thomas, | 2 00 |
| **IOWA.** | |
| Dr. H. Fiske, | $2 00 |

